# Poster 2016: The effect and value of sublingual immunotherapy: a patient survey

**DOI:** 10.1186/1939-4551-7-S1-P26

**Published:** 2014-02-03

**Authors:** Mary S Morris, Emma Killoran, Jeffrey Kessler, Moira Killoran, Amanda Lowery

**Affiliations:** 1 Allergy Associates of La Crosse, WI, USA; 2 Allergychoices, USA

## Background

The purpose of this study was to measure the health status, quality of life, and perceived value of sublingual immunotherapy (SLIT) treatment from patients of Allergy Associates of La Crosse (AAOL).

## Methods

A survey was sent to a random sample of 1,400 patients obtained from the AAOL newsletter database of 4,500 patients. The 20 question survey assessed patient demographics, perceived value of treatment, medication use, health and utilization ratings, compliance, school/work attendance, hospitalizations and unplanned physician visits and health related measures such as energy, sleep, and emotional well-being. Data was analyzed using SPSS by the research team.

## Results

299 patients completed the survey. The average age of respondent was 36-55 years old. The average distance traveled to the clinic was 244 miles. 92 percent of the patients reported that they had or planned to complete treatment with 81 percent reporting compliance all or most of the time. Patients reported an average improvement in their general health status of 90 percent (t=28.2, p<0.0001) with an average prior health rating of 2.12 and an average current health rating of 4.1 (1=very poor, 5=very good). ER visits were significantly reduced (p<0.0001) and were eliminated completely for 37 patients that had reported ER visits prior to treatment. Emotional wellbeing measures also improved. 77 percent reported an improvement in sleep and 83 percent reported an improvement in daily energy. 78 percent showed improvement in productivity at school/work. Aspects of emotional wellbeing also improved. Value of treatment (was SLIT worth the investment given the results) was assessed on a scale of 1-5 with 1 being not at all and 5 being very much so. 70 percent of patients reported a perceived value of very much so with an average response of 4.56. There was no correlation between gender or distance traveled and perceived value of treatment.

## Conclusions

The survey demonstrated a high level of efficacy and value of treatment for patients treated with SLIT at AAOL. Patients reported improvement in health and quality of life. The high perceived value of treatment is a testament to the worth of SLIT for allergy treatment as patients reported the results were worth the investment required. The results of this survey support the use of SLIT in clinical treatment of allergies.

